# Pituitary Volume and Socio-Cognitive Functions in Individuals at Risk of Psychosis and Patients With Schizophrenia

**DOI:** 10.3389/fpsyt.2018.00574

**Published:** 2018-11-09

**Authors:** Tsutomu Takahashi, Yuko Higuchi, Yuko Komori, Shimako Nishiyama, Yoichiro Takayanagi, Daiki Sasabayashi, Mikio Kido, Atsushi Furuichi, Yumiko Nishikawa, Mihoko Nakamura, Kyo Noguchi, Michio Suzuki

**Affiliations:** ^1^Department of Neuropsychiatry, University of Toyama Graduate School of Medicine and Pharmaceutical Sciences, Toyama, Japan; ^2^Department of Radiology, University of Toyama Graduate School of Medicine and Pharmaceutical Sciences, Toyama, Japan

**Keywords:** at-risk mental state, schizophrenia, pituitary gland, HPA axis, working memory

## Abstract

**Objectives:** Increased pituitary volume, which probably reflects hypothalamic-pituitary-adrenal (HPA) hyperactivity, has been reported in patients with schizophrenia and individuals at risk of psychosis. On the basis of potential role of abnormal HPA axis function on cognitive impairments in psychosis, we aimed to examine possible relations between the pituitary volume and socio-cognitive impairments in these subjects.

**Methods:** This magnetic resonance imaging study examined the pituitary gland volume in 38 subjects with at-risk mental state (ARMS) [of whom 4 (10.5%) exhibited the transition to schizophrenia], 63 patients with schizophrenia, and 61 healthy controls. Social and cognitive functions of the ARMS and schizophrenia groups were assessed using the Brief Assessment of Cognition in Schizophrenia (BACS), the Schizophrenia Cognition Rating Scale (SCoRS), and the Social and Occupational Functioning Assessment Scale (SOFAS).

**Results:** Both the ARMS and schizophrenia groups had a significantly larger pituitary volume compared to controls. In the schizophrenia group, the pituitary volume was negatively associated with the BACS working memory score. No association was found between the pituitary volume and clinical variables (medication, symptom severity) in either clinical group.

**Conclusion:** Our findings support the notion of common HPA hyperactivity in the ARMS and schizophrenia groups, but abnormal HPA axis function may contribute differently to cognitive deficits according to the illness stages of schizophrenia.

## Introduction

Neuroendocrine studies in schizophrenia (1, 2) and clinical high-risk subjects for developing psychosis [i.e., at-risk mental state; ARMS (3, 4)] (5–7) have reported hyperactivity of the hypothalamic-pituitary-adrenal (HPA) axis, which mediates the stress response by governing the release of steroids (e.g., cortisol) and also regulates a number of physiological and neurobehavioral processes (e.g., immunity, fertility, anxiety, and cognitive functioning) (8, 9), implying the role of hormonal dysregulation during the course of psychosis. Previous magnetic resonance imaging (MRI) studies in schizophrenia and related psychoses have generally reported enlarged volume of the pituitary gland, an integral part of the HPA axis, prior to psychosis onset (10, 11), along with ongoing expansion early in the course of schizophrenia (12, 13), which was associated with the emergence of psychosis and the early course of clinical symptoms (14, 15). However, some discrepant findings, such as an even smaller pituitary volume in antipsychotic-naïve schizophrenia patients with recent onset (16) or normal pituitary volume both in patients with first-episode schizophrenia and individuals with ARMS (7), have also been reported. Thus, pituitary findings in schizophrenia and high-risk subjects remain elusive and further studies will be needed to clarify the role of HPA axis abnormality and its relation to clinical characteristics in these subjects.

Cognitive impairments, particularly in memory and executive function, are a core feature of psychosis that exist during first-episode (17, 18) or even before psychosis onset (19, 20), and are also associated with poor functional outcome (21, 22). Previous neuroendocrine studies have demonstrated that these cognitive impairments (especially memory deficits) are at least partly due to abnormal HPA axis function, as indexed by an elevated diurnal cortisol level and/or blunted cortisol awaking response, in both schizophrenia (23, 24) and high-risk individuals (25). However, it is also noted that different mechanisms may contribute to distinct HPA axis abnormalities for vulnerability and onset of psychosis (25) and that the relationship between the HPA axis and memory functioning may differ at different illness stages (26). To our knowledge, it is unknown whether the pituitary volume in schizophrenia, which probably reflects HPA axis functioning, is associated with cognitive function and whether their relations differ during the course of the illness.

The present MRI study aimed to investigate the pituitary volume in individuals with ARMS and patients with schizophrenia in comparison with healthy subjects and to examine whether pituitary volume was related to neurocognitive measures and social functioning in these subjects. On the basis of our previous MRI study in an independent sample of early psychosis (11), as well as the potential role of HPA axis dysregulation in modulating cognitive function in patients with psychosis (24, 25), we predicted enlarged pituitary volume in both the ARMS and schizophrenia groups, which could be partly related to cognitive impairments in these subjects.

## Materials and methods

### Participants

Thirty-eight individuals with ARMS, 63 schizophrenia patients, and 61 healthy subjects were included in this study. Recruitment strategies for the study participants in our department have been described in detail elsewhere (27, 28).

Briefly, the individuals with ARMS, who had no previous episode of overt psychosis, were recruited from the Consultation Support Service in Toyama (CAST), a specialized clinical setting for young people (aged 15–30 years) at risk for psychosis (29). All subjects were categorized as the attenuated psychotic symptoms (APS) group (4) according to the Japanese version of the CAARMS (30). Comorbid DSM-IV-TR Axis I diagnoses (31) were anxiety disorders (*N* = 9), pervasive developmental disorders (*N* = 6), depressive disorders (*N* = 6), schizotypal personality disorders (*N* = 6), adjustment disorders (*N* = 1), or dissociative disorders (*N* = 1). Four subjects had no axis I diagnosis. They were prospectively followed up regularly at outpatient clinics of the Department of Neuropsychiatry of Toyama University Hospital; four (10.5%) of the ARMS group developed schizophrenia during clinical follow-up (mean follow-up period = 896.1 ± 841.6 days, median = 581.5). Medication status and other clinical data are summarized in Table 1. They were also receiving benzodiazepines (*N* = 6), antidepressants (*N* = 4), and/or tandospirone (*N* = 1) at the time of scanning.

**Table 1:** Demographic/clinical data, socio-cognitive functions, and brain measures in the ARMS, schizophrenia, and control subjects.

	**Controls**	**ARMS**	**Sz**	**Group difference**
	**(*N* = 61)**	**(*N* = 38)**	**(*N* = 63)**
Age	25.6 ± 3.2	18.4 ± 3.9	28.0 ± 9.4	*F*_(2, 159)_ = 27.01, *p* < 0.001; ARMS < Contols, Sz
Male/female	32/29	24/14	29/34	Chi-square = 2.79, *p* = 0.248
Height (cm)	166.0 ± 8.3	165.3 ± 9.0	163.2 ± 8.4	*F*_(2, 159)_ = 1.80, *p* = 0.168
JART-IQ	110.2 ± 5.9	98.0 ± 10.2	99.5 ± 9.7	*F*_(2, 159)_ = 32.91, *p* < 0.001; ARMS, Sz < Controls
Handedness (right/mixed/left)	40/15/6	22/12/4	52/9/2	Fisher's exact test, *p* = 0.064
SES	6.3 ± 0.9	3.2 ± 1.4	4.2 ± 1.4	*F*_(2, 159)_ = 82.61, *p* < 0.001; ARMS < Sz < Controls
Parental SES	5.9 ± 0.9	4.8 ± 0.9	4.8 ± 1.4	*F*_(2, 158)_ = 17.86, *p* < 0.001; ARMS, Sz < Controls
Onset age (years)	–	–	22.4 ± 7.4	–
Illness duration (years)	–	–	5.5 ± 6.0	–
Medication dose (HPD equiv., mg/day)	–	2.0 ± 1.6 (*N* = 11)	11.3 ± 7.8 (*N* = 51)	*F*_(1, 59)_ = 15.15, *p* < 0.001; ARMS < Sz
Medication type (atypical/typical/mixed)	–	9/1/1	45/1/5	Fisher's exact test, *p* = 0.372
Duration of medication (years)	–	0.7 ± 1.3 (*N* = 14)	5.2 ± 6.2 (*N* = 53)	*F*_(1, 64)_ = 0.05, *p* = 0.820
Serum prolactin level (ng/mL)	–	14.5 ± 13.9 (*N* = 27)	47.6 ± 73.4 (*N* = 45)	*F*_(1, 69)_ = 5.37, *p* = 0.023; ARMS < Sz
PANSS positive	–	11.4 ± 3.6	13.9 ± 5.6	*F*_(1, 98)_ = 5.20, *p* = 0.024; ARMS < Sz
PANSS negative	–	15.4 ± 6.7	16.3 ± 5.6	*F*_(1, 98)_ = 3.97, *p* = 0.049; not significant (*post-hoc* test)
PANSS general	–	30.4 ± 8.1	31.0 ± 9.7	*F*_(1, 98)_ = 1.38, *p* = 0.243
SOFAS^a^	–	52.2 ± 10.8	48.2 ± 13.9	*F*_(1, 97)_ = 4.52, *p* = 0.036: not significant (*post-hoc* test)
SCoRS global rating score^a^	–	5.4 ± 2.4	5.2 ± 2.5	*F*_(1, 97)_ = 0.49, *p* = 0.487
BACS subdomain z-scores				Group x domain interaction, *F*_(5, 495)_ = 5.64, *p* < 0.001
Verbal memory	–	−0.9 ± 1.6	−1.3 ± 1.4	*p* = 0.933
Working memory	–	−0.8 ± 1.4	−1.0 ± 1.4	*p* = 1.000
Motor function	–	−0.8 ± 1.4	−1.9 ± 1.5	*p* = 0.009; Sz < ARMS
Verbal fluency	–	−1.0 ± 1.6	−0.8 ± 1.1	*p* = 1.000
Attention and processing speed	–	−0.3 ± 1.3	−1.4 ± 1.5	*p* = 0.013; Sz < ARMS
Executive function	–	−0.5 ± 1.3	−0.8 ± 1.6	*p* = 1.000
Pituitary volume (mm^3^)	599 ± 112	687 ± 134	739 ± 150	*F*_(2, 154)_ = 18.62, *p* < 0.001; Contols < ARMS, Sz
Intracranial volume (ml)	1,459 ± 126	1,408 ± 127	1,441 ± 149	*F*_(2, 158)_ = 1.25, *p =* 0.288^b^
Total gray matter volume (ml)	754 ± 55	749 ± 66	704 ± 102	*F*_(2, 154)_ = 6.60, *p =* 0.002; Sz < ARMS, Controls

*Values represent Means ± SDs unless otherwise stated*.

*ARMS, at risk mental state; BACS, Brief Assessment of Cognition in Schizophrenia; JART, Japanese version of National Adult Reading Test; HPD, haloperidol; PANSS, Positive and Negative Syndrome Scale; SCoRS, Schizophrenia Cognition Rating Scale; SES, socioeconomic status; SOFAS, Social and Occupational Functioning Assessment Scale; Sz, schizophrenia*.

a *Data missing for one schizophrenia patient*.

b *Age was used as a covariate*.

The schizophrenia patients fulfilling the DSM-IV-TR criteria (31) were recruited from inpatient and outpatient clinics of Toyama University Hospital. They were diagnosed based on information obtained from a clinical assessment using the Structured Clinical Interview for DSM-IV Axis I Disorders Patient Edition (SCID-I/P) (32), a detailed chart review, as well as the clinical symptoms rated at the time of scanning. Medication and other clinical data are summarized in Table 1. At the time of scanning, experienced psychiatrists rated the clinical symptoms of the ARMS and schizophrenia subjects using the Positive and Negative Syndrome Scale (PANSS) (33).

The healthy controls, who were screened for psychiatric illness using the SCID-I Non-patient Edition (32), were recruited from hospital staff, members of the local community, and university students. They were also screened using a questionnaire consisting of 19 items concerning their personal (17 items; including a history of obstetric complications, serious head injury, seizures, neurological illness, impaired thyroid function, hypertension, diabetes, and substance abuse) and family (2 items) histories of illness. Subjects with family history of psychiatric illness among their first-degree relatives were excluded.

All participants in this study were physically healthy at the time of the study and none had a lifetime history of serious head trauma, neurological illness, serious medical or surgical illness, substance abuse, or steroid use. Handedness (34), personal and parental socioeconomic status (SES) (35), and IQ estimated using the Japanese version of the National Adult Reading Test (JART) (36) were also evaluated. None of the participants was pregnant or taking exogenous estrogens at the time of the study, but estrogen levels and menstrual cycle in female subjects were not assessed. Serum prolactin levels at the time of scanning were available for 27 ARMS and 45 schizophrenia subjects. While we previously reported the pituitary volume in early psychosis using 1.5T MRI data (11), this was our first study of the pituitary gland using independent 3T MRI data. This study received approval from the Committee on Medical Ethics of Toyama University (No. 25-7). Written informed consent was obtained from all subjects in accordance with the Declaration of Helsinki. When participants were under the age of 20, written consent was also obtained from the parent/guardian.

### MRI acquisition and data processing

Magnetic resonance images were obtained by utilizing a 3-T Magnetom Verio (Siemens Medical System, Inc., Erlangen, Germany) with a 12-channel head coil. A three-dimensional magnetization-prepared rapid gradient echo (MPRAGE) sequence yielded 176 contiguous T1-weighted slices of 1.2-mm thickness in the sagittal plane. The imaging parameters were: repetition time = 2,300 ms; echo time = 2.9 ms; flip angle = 9°; field of view = 256 mm; and matrix size = 256 × 256 pixels. The voxel size was 1.0 × 1.0 × 1.2 mm.

The image data were then processed on a Macintosh computer (Apple Inc., California, USA) using Dr. View software (Infocom, Tokyo, Japan) (11, 37, 38). Brain images were realigned in three dimensions to standardize for differences in head tilt during image acquisition and were then reconstructed into entire contiguous coronal images, with a 1-mm thickness, perpendicular to the anterior commissure-posterior commissure line. The signal-intensity histogram distributions from the T1-weighted images across the whole cerebrum were then used to semi-automatically segment the voxels into brain tissue components and cerebrospinal fluid. The intracranial volume (ICV) (i.e., the sum of gray matter, white matter, and CSF volumes) was estimated using SPM 12 (https://www.fil.ion.ucl.ac.uk/spm/software/spm12/) to correct for differences in head size (39); the groups did not significantly differ in their ICV volumes, but the schizophrenia group had a significantly smaller total gray matter volume as compared with other groups (Table 1).

### Pituitary measurements

**Figure 1:**
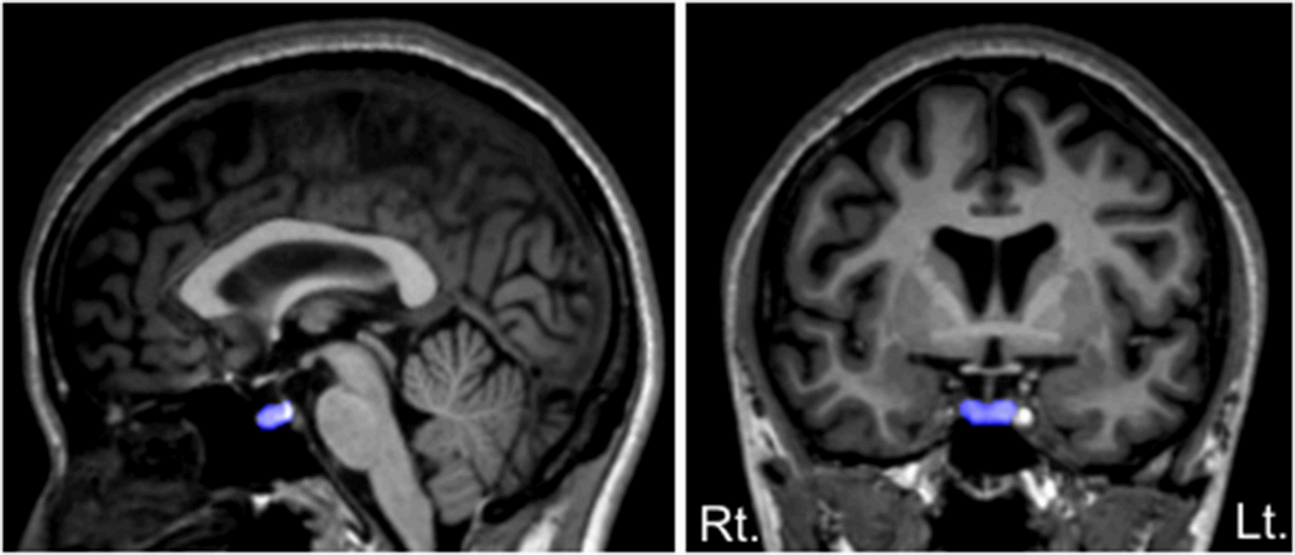
Sagittal **(left)** and coronal **(right)** views of the pituitary gland manually traced in this study. The pituitary stalk was excluded from the tracings, but we included a posterior bright spot, corresponding to the posterior pituitary (the intensity of which is thought to reflect vasopressin concentrations).

As described in detail elsewhere (11, 37, 38), the volume of the pituitary gland was manually traced on 1.0-mm consecutive coronal slices based on a method used by Garner et al. (40). Briefly, we traced around the usually well-defined borders of the anterior and posterior pituitary: the diaphragma sellae, superiorly; the sphenoid sinus, inferiorly; and the cavernous sinuses, bilaterally (Figure 1).

All measurements were carried out by one rater (TT) without knowledge of the subject's identity, gender or diagnosis. Inter- (TT and DS) and intra-rater intraclass correlation coefficients in a subset of 10 randomly selected brains were 0.82 and 0.86, respectively.

### Assessment of socio-cognitive functions

Socio-cognitive functions were assessed using the same method as in our previous studies of olfactory functioning (27) and quality of life (28). All of these assessments were administered by an experienced psychologist (YK) at the time of scanning.

Briefly, the cognitive functioning was assessed using the Japanese version (41) of the Brief Assessment of Cognition in Schizophrenia (BACS) (42), which includes six cognitive domains (verbal memory, working memory, motor speed, verbal fluency, attention, and executive function). The primary measure from each test of the BACS was standardized by creating z-scores, whereby the mean score of Japanese healthy controls was set to zero and the standard deviation set to one (43). The study participants were also administered the Schizophrenia Cognition Rating Scale (SCoRS), an interview-based measure of cognitive abilities related to daily-living functioning (44). Based on three different sources (i.e., an interview with the patient, an interview with the caregiver(s), and the interviewer's rating), the rater (interviewer) assigned the SCoRS global rating score (range 1–10, higher ratings indicate greater impairment in daily living skills). Social functioning was assessed using the Social and Occupational Functioning Assessment Scale (SOFAS) (45), which corresponds to the social functioning domains of the Global Assessment of Functioning Scale in the DSM-IV (46). The scores range from 0 to 100, with higher scores indicating better functioning.

### Statistical analysis

Group differences in the demographic data were assessed by using one-way analysis of variance (ANOVA) or chi-square test. Clinical variables and social/cognitive functions were compared using the analysis of covariance (ANCOVA) with age as a covariate, because a significant group difference in age could affect these variables.

Group difference in the absolute pituitary volume was analyzed using ANCOVA with ICV and age as covariates, with diagnosis and gender as between-subject factors. Then, the schizophrenia patients were divided into first-episode (illness duration ≤ 12 months, 8 males and 9 females) and chronic (illness duration ≥ 36 months, 17 males and 21 females) subgroups; the pituitary volume was compared with the same ANCOVA model but with the subgroups (first-episode, chronic) and gender as between-subject factors. The absolute pituitary volume of neuroleptic-free patients (27 ARMS and 12 schizophrenia patients) and those who were receiving antipsychotic medication (11 ARMS and 51 schizophrenia patients) was also analyzed by ANCOVA. *Post-hoc* Scheffé's tests were carried out to follow up these analyses. The study findings remained essentially the same even when we included medication dose and duration as the covariates.

Spearman's rank correlations were calculated to examine relationships between relative pituitary volume [(absolute volume / ICV) × 100] and the clinical/socio-cognitive variables. Statistical significance was defined as *p* < 0.05.

## Results

### Demographic, clinical and socio-cognitive characteristics

Table 1 shows the sample characteristics of the study participants. The groups did not differ in gender and height, but there were group differences in age, IQ, and parental/personal SES.

The individuals with ARMS were characterized by lower amounts of antipsychotics, less severe positive symptoms, and higher BACS measures compared with the patients with schizophrenia. However, the first-episode and chronic schizophrenia subgroups did not differ in terms of the symptom severity or socio-cognitive measures.

### Pituitary gland volume

**Figure 2:**
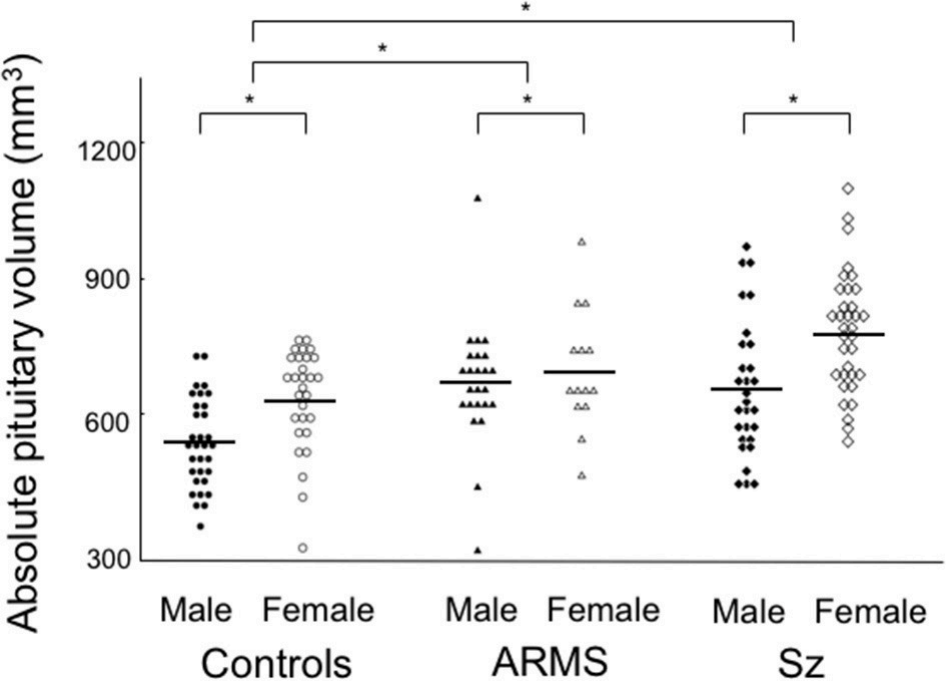
Absolute pituitary gland volume in the subjects with at-risk mental state (ARMS), patients with schizophrenia (Sz), and healthy controls. Horizontal lines indicate mean values. *post-hoc* test: *p < 0.01.

ANCOVA of the pituitary volume demonstrated significant main effects for diagnosis (Table 1) and gender (*F* = 16.83; *df* = 1, 154; *p* < 0.001), but no diagnosis-by-gender interaction was found (*F* = 2.14; *df* = 2, 154; *p* = 0.122). *Post-hoc* analyses showed that the schizophrenia (*p* < 0.001) and ARMS (*p* = 0.003) groups had significantly larger pituitary volumes compared to controls (Figure 2) and there was a significant gender difference in pituitary size (female > male, *p* < 0.001). The pituitary volume did not differ between the ARMS and schizophrenia groups (*p* = 0.247).

The ARMS individuals who later developed schizophrenia had a comparable pituitary volume (*N* = 4; mean = 691 mm^3^, *SD* = 58) with those who did not (*N* = 34; mean = 687 mm^3^, *SD* = 141). The first episode (mean = 747 mm^3^, *SD* = 156) and chronic (mean = 740 mm^3^, *SD* = 150) schizophrenia groups did not significantly differ for pituitary volume (ANCOVA, *F* = 0.03; *df* = 1, 49; *p* = 0.863). The patients treated with antipsychotics (mean = 750 mm^3^, *SD* = 145) had a larger pituitary volume than antipsychotic-free patients (mean = 671 mm^3^, *SD* = 135) (ANCOVA, *F* = 4.56; *df* = 1, 95; *p* = 0.035), while the pituitary volume in these antipsychotic-free patients was significantly larger than controls (ANCOVA, *F* = 8.67; *df* = 1, 94; *p* = 0.004).

### Correlational analyses

**Figure 3:**
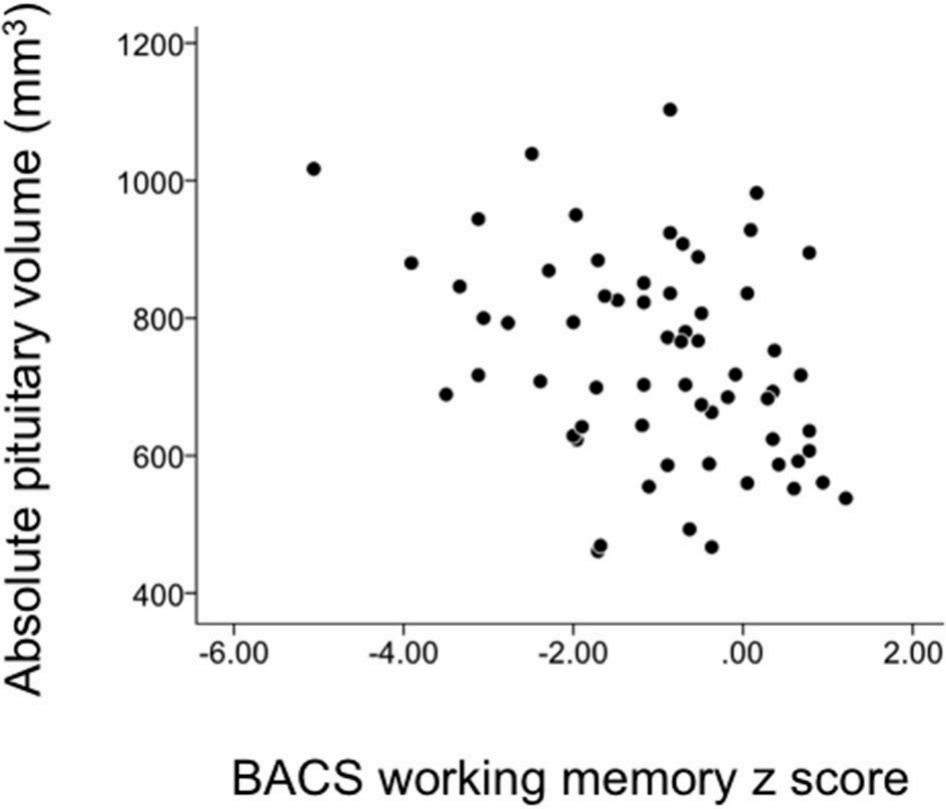
Correlation between the pituitary volume and the Brief Assessment of Cognition in Schizophrenia (BACS) working memory score in schizophrenia patients.

The relative pituitary volume was negatively correlated with age only in healthy controls (*rho* = −0.403, *p* = 0.001). There was no significant relation between the pituitary volume and clinical variables, but the BACS working memory score in schizophrenia was negatively correlated with pituitary volume (Table 2 and Figure 3). This correlation survived Bonferroni's correction for multiple comparisons [28 comparisons; *p* < 0.00179 (0.05/28)] (Table 2). The correlation between the pituitary volume and working memory, which did not change even when we used Pearson's partial correlation coefficients controlling for age (*r* = −0.39, *p* = 0.00177) or medication dose and duration (*r* = −0.39, *p* = 0.00171), was more evident in first-episode (*rho* = −0.56, *p* = 0.020) than in chronic (*rho* = −0.31, *p* = 0.057) patients. For the validation purpose, we then assessed the independent contribution of all demographic/clinical variables except for other BACS subdomain scores (Table 1) to predicting the BACS working memory score in schizophrenia by using stepwise regression analysis; the working memory score was significantly predicted only by the pituitary volume (Beta = −0.316, *t* = −2.64, *p* = 0.011) and PANSS negative score (Beta = −0.269, *t* = −2.24, *p* = 0.029) (Adjusted *R*^2^ = 0.185).

**Table 2:** Correlations between the pituitary volume and clinical/socio-cognitive variables.

	**ARMS**		**Schizophrenia**	
	***rho***	***p***	***rho***	***p***
Onset age (years)	–	–	−0.17	0.174
Illness duration	–	–	0.02	0.892
Medication dose	0.06	0.730	0.17	0.190
Duration of medication	−0.01	0.967	0.11	0.382
SOFAS	0.15	0.373	−0.16	0.205
PANSS positive	0.13	0.437	0.12	0.349
PANSS negative	0.08	0.627	−0.01	0.971
PANSS general	−0.01	0.952	−0.02	0.852
SCoRS global rating score	−0.07	0.672	0.14	0.276
BACS z-scores			
Verbal memory	0.29	0.081	−0.26	0.037
Working memory	−0.05	0.782	−0.39	0.00176^a^
Motor function	−0.02	0.912	−0.11	0.412
Verbal fluency	0.07	0.660	0.12	0.362
Attention and processing speed	0.25	0.136	−0.10	0.440
Executive function	0.10	0.548	−0.27	0.034

*ARMS, at risk mental state; BACS, Brief Assessment of Cognition in Schizophrenia; PANSS, Positive and Negative Syndrome Scale; SCoRS, Schizophrenia Cognition Rating Scale; SOFAS, Social and Occupational Functioning Assessment Scale*.

a *Significant after Bonferroni's correction for multiple comparisons [28 comparisons; p < 0.00179 (0.05/28)]*.

We also examined the possible relation between the relative pituitary volume and serum prolactin levels in a subsample of 72 (27 ARMS and 45 schizophrenia) subjects, which showed a significant positive correlation (*rho* = 0.293, *p* = 0.012). The correlation between the pituitary volume and working memory in schizophrenia was not significant when we used prolactin level as a controlling factor (*rho* = −0.23, *p* = 0.122).

## Discussion

To our knowledge, this is the first MRI study to demonstrate a significant correlation between the pituitary volume and cognitive impairments in schizophrenia. In the present study, we replicated our previous finding of enlarged pituitary volume in both the ARMS and schizophrenia groups (11) in an independent cohort. The pituitary enlargement was significantly associated with working memory deficits, but not with clinical or other socio-cognitive measures, specifically in schizophrenia patients especially for the first-episode subgroup. These findings may reflect HPA hyperactivity as a possible indicator of vulnerability to psychosis, but also support the potential role of distinct HPA axis abnormalities in the cognitive impairments in different illness stages (25, 26).

Our pituitary findings are generally in line with previous MRI studies in clinical high-risk subjects; the individuals who later develop psychosis may exhibit pituitary expansion prior to psychosis onset (10, 40), but those without psychosis onset also have similar pituitary changes (10, 11). Although some high-risk studies did not replicate these findings (7, 47), probably due to small sample sizes and/or differences in various influencing factors as described below, pituitary expansion reported in psychosis is thought to reflect HPA hyperactivity and a subsequent increase in the size and number of corticotrophs, whereas chronic HPA activation could cause pituitary atrophy by reducing the function of the cells producing other pituitary hormones (15, 48). Thus, the pituitary volume in the course of psychosis likely reflects state-related HPA axis dysregulation, which is associated with illness stages and symptom severity (15), antipsychotic medication (2, 49), demographic characteristics [e.g., age, gender (50, 51)], and other mediating factors. Indeed, the present results supported that the pituitary gland is especially sensitive to prolactin-elevating antipsychotics (12, 52), while the effect of medication alone could not explain pituitary expansion in the antipsychotic-free subsample. On the other hand, despite a significant group difference in the medication status and symptom severity (especially for positive psychotic symptoms), we demonstrated that the pituitary gland was expanded to a similar degree in high-risk subjects as in schizophrenia patients, suggesting that distress related to prodromal symptomatology or impaired role functioning could activate the stress response even without florid psychosis.

One major finding of this study was that pituitary expansion in schizophrenia significantly correlated with working memory deficits, while such a correlation was not observed in the ARMS group. Consistent with previous studies (19, 20), the schizophrenia patients showed global cognitive deficits, with those for some domains (e.g., attention and processing speed) being more severe compared to high-risk subjects. Among these deficits, working memory impairment, which exists prior to the onset of psychosis (19, 20), is considered a central cognitive impairment in schizophrenia that is associated with a range of clinical characteristics [e.g., both positive (53) and negative (54) symptomatology and social deficits (55)]. While candidate neural circuits for working memory dysfunction in schizophrenia include the frontal-striatal-thalamic systems, particularly those involving the dorsolateral prefrontal cortex (54, 56), there is also neuroendocrine evidence that abnormal HPA axis function (i.e., flattened diurnal cortisol slope) is associated with working memory deficits in the early stages of psychosis (57) and that higher levels of dehydroepiandosterone (DHEA), an HPA-related hormone that counteracts the negative effects of cortisol in the brain (58), are associated with better working memory performance in schizophrenia (59). However, it is also suggested that each of the working memory components (e.g., the temporary systems and central executive system) may be differently impaired in psychosis (60) and that cortisol is linked with memory function in two different ways: (1) directly, by acutely disrupting working memory and short-term recall, and (2) indirectly, through the effects of persistent cortisol elevation on hippocampal integrity (23). Thus, the relation between the HPA axis dysregulation and memory deficits in psychosis is complex and may differ according to illness stages (26). Our findings of a significant relation between pituitary expansion and working memory impairment, especially in first-episode schizophrenia, but not in ARMS individuals, may reflect acute HPA hyperactivity that emerges only proximally to psychosis onset (25).

We note several limitations in this study. First, the sample size of our ARMS cohort (especially those who developed psychosis) was relatively small and their clinical follow-up periods were short (<12 months) for a substantial number of cases (*N* = 14). We therefore could not reliably examine the relationship between the pituitary volume or cognitive measures and later transition into psychosis. In addition, the ARMS group in this study was younger than the other groups. Although we statistically controlled for age differences, pituitary findings and their relation to clinical characteristics should be further tested in a larger, well-defined high-risk cohort in comparison with age-matched controls. Second, we did not assess pituitary function in this study. Although our findings regarding pituitary expansion are thought to reflect HPA axis dysregulation, a recent study of multiple measures of HPA axis function (7) did not find any association between the pituitary volume and cortical measures in high-risk and first-episode schizophrenia patients. The present study replicated the gender difference in the pituitary volume (female > male), probably due to different endogenous estrogen levels (51), but we did not assess estrogen levels. Thus, further assessment of both pituitary volume and hormonal levels (e.g., cortisol, DHEA, and estrogen) will be needed. Third, we did not assess other brain regions closely associated with memory function (e.g., hippocampus), representing a major limitation of the study. In this study, the correlation between the pituitary volume and working memory remained essentially the same even when controlling for total gray matter volume (*r* = −0.38, *p* = 0.002), suggesting no major contribution of gray matter changes to the pituitary-cognition relationship. However, future studies should conduct more comprehensive assessment to investigate potential neural underpinnings of working memory deficits in schizophrenia. Finally, most schizophrenia patients and 11 high-risk subjects were receiving antipsychotics at the time of the study, which could have affected both pituitary volume (2) and cognitive function (61). Although we did not find a direct relation between the medication (dose, duration) and pituitary volume, BACS subscale scores, or their relationships for either the ARMS or schizophrenia groups, our results suggested that serum prolactin level could be a confounding factor for our main findings. While schizophrenia patients frequently show hyperprolactinemia as a consequence of antipsychotic treatment, several studies have reported an elevated prolactin level and its relation to cognitive function independent of medication (62). Thus, possible role of prolactin on the relation between the pituitary volume and cognitive impairments should be further tested in an antipsychotic-naïve cohort.

In summary, the present study demonstrated that clinical high-risk subjects for psychosis exhibit enlargement of the pituitary gland similar to that observed in established schizophrenia, possibly reflecting a common vulnerability. On the other hand, our findings demonstrated that the pituitary volume may be specifically associated with working memory deficits during the first-episode of schizophrenia. These findings may support the potential role of distinct HPA axis abnormalities that contribute to the cognitive impairments in different illness stages, but future studies should also examine hormone levels to understand the role of HPA functioning during the course of psychosis.

## Author contributions

MS, YH, and TT conceived the idea and methodology of the study. TT conducted the statistical analyses and wrote the manuscript. SN, DS, MN, and YN recruited subjects and were involved in clinical and diagnostic assessments. TT, DS, and MK analyzed the MRI data. YK assessed the socio-cognitive functions of the study participants. KN provided technical support for MRI scanning and data processing. AF, YN, and MN managed the MRI and clinical data. MS, YH, and YT contributed to the writing and editing of the manuscript. All authors contributed to and have approved the final manuscript.

### Conflict of interest statement

The authors declare that the research was conducted in the absence of any commercial or financial relationships that could be construed as a potential conflict of interest.

## References

[1] Phillips LJ McGorry PD Garner B Thompson KN Pantelis C Wood SJ. Stress, the hippocampus and the hypothalamic-pituitary-adrenal axis:implications for the development of psychotic disorders. Aust N Z J Psychiatry (2006) 40:725–41. 10.1080/j.1440-1614.2006.01877.x 16911747

[2] Walker E Mittal V Tessner K. Stress and the hypothalamic pituitary adrenal axis in the developmental course of schizophrenia. Ann Rev Clin Psychol. (2008) 4:189–216. 10.1146/annurev.clinpsy.4.022007.141248 18370616

[3] Yung AR Phillips LJ McGorry PD Treating Schizophrenia in the Prodromal Phase. London:Taylor & Francis (2004).

[4] Yung AR Yuen HP McGorry PD Phillips LJ Kelly D Dell'Olio M. Mapping the onset of psychosis:the comprehensive assessment of at-risk mental states. Aust N Z J Psychiatry (2005) 39:964–71. 10.1080/j.1440-1614.2005.01714.x 16343296

[5] Walker EF Brennan PA Esterberg M Brasfield J Pearce B Compton MT. Longitudinal changes in cortisol secretion and conversion to psychosis in at-risk youth. J Abnorm Psychol. (2010) 119:401–8. 10.1037/a0018399 20455612 PMC3052257

[6] Aiello G Horowitz M Hepgul N Pariante CM Mondelli V. Stress abnormalities in individuals at risk for psychosis:a review of studies in subjects with familial risk or with “at risk” mental state. Psychoneuroendocrinology (2012) 37:1600–13. 10.1016/j.psyneuen.2012.05.003 22663896

[7] Nordholm D Rostrup E Mondelli V Randers L Nielsen MØ Wulff S. Multiple measures of HPA axis function in ultra high risk and first-episode schizophrenia patients. Psychoneuroendocrinology (2018) 92:72–80. 10.1016/j.psyneuen.2018.03.015 29635174

[8] Franz CE O'Brien RC Hauger RL Mendoza SP Panizzon MS Prom-Wormley E. Cross-sectional and 35-year longitudinal assessment of salivary cortisol and cognitive functioning:the Vietnam Era twin study of aging. Psychoneuroendocrinology (2011) 36:1040–52. 10.1016/j.psyneuen.2011.01.002 21295410 PMC3130089

[9] DeMorrow S. Role of the hypothalamic-pituitary-adrenal axis in health and disease. Int J Mol Sci. (2018) 19:986. 10.3390/ijms19040986 29587417 PMC5979578

[10] Büschlen J Berger GE Borgwardt SJ Aston J Gschwandtner U Pflueger MO. Pituitary volume increase during emerging psychosis. Schizophr Res. (2011) 125:41–8. 10.1016/j.schres.2010.09.022 21074369

[11] Takahashi T Nakamura K Nishiyama S Furuichi A Ikeda E Kido M. Increased pituitary volume in subjects at risk for psychosis and patients with first-episode schizophrenia. Psychiatry Clin Neurosci. (2013) 67:540–8. 10.1111/pcn.12093 24102999

[12] MacMaster FP El-Sheikh R Upadhyaya AR Nutche J Rosenberg DR Keshavan M. Effect of antipsychotics on pituitary gland volume in treatment-naïve first-episode schizophrenia:a pilot study. Schizophr Res. (2007) 92:207–10. 10.1016/j.schres.2007.01.022 17337162

[13] Takahashi T Zhou SY Nakamura K Tanino R Furuichi A Kido M. Longitudinal volume changes of the pituitary gland in patients with schizotypal disorder and first-episode schizophrenia. Prog Neuropsychopharmacol Biol Psychiatry (2011) 35:177–83. 10.1016/j.pnpbp.2010.10.023 21044655

[14] Nordholm D Krogh J Mondelli V Dazzan P Pariante C Nordentoft M. Pituitary gland volume in patients with schizophrenia, subjects at ultra high-risk of developing psychosis and healthy controls:a systematic review and meta-analysis. Psychoneuroendocrinology (2013) 38:2394–404. 10.1016/j.psyneuen.2013.06.030 23890984

[15] Takahashi T Suzuki M Brain morphologic changes in early stages of psychosis:implications for clinical application and early intervention. Psychiatry Clin Neurosci. (2018) 72:556–71. 10.1111/pcn.12670 29717522

[16] Upadhyaya AR El-Sheikh R MacMaster FP Diwadkar VA Keshavan MS. Pituitary volume in neuroleptic-naïve schizophrenia:a structural MRI study. Schizophr Res. (2007) 90:266–73. 10.1016/j.schres.2006.09.033 17187962

[17] Mesholam-Gately RI Giuliano AJ Goff KP Faraone SV Seidman LJ. Neurocognition in first-episode schizophrenia:a meta-analytic review. Neuropsychology (2009) 23:315–36. 10.1037/a0014708 19413446

[18] Aas M Dazzan P Mondelli V Melle I Murray RM Pariante CM. A systematic review of cognitive function in first-episode psychosis, including a discussion on childhood trauma, stress, and inflammation. Front Psychiatry (2014) 4:182. 10.3389/fpsyt.2013.00182 24409157 PMC3884147

[19] Fusar-Poli P Deste G Smieskova R Barlati S Yung AR Howes O. Cognitive functioning in prodromal psychosis:a meta-analysis. Arch Gen Psychiatry (2012) 69:562–71. 10.1001/archgenpsychiatry.2011.1592 22664547

[20] De Herdt A Wampers M Vancampfort D De Hert M Vanhees L Demunter H Neurocognition in clinical high risk young adults who did or did not convert to a first schizophrenic psychosis:a meta-analysis. Schizophr Res. (2013) 149:48–55. 10.1016/j.schres.2013.06.017 23830855

[21] Lin A Wood SJ Nelson B Brewer WJ Spiliotacopoulos D Bruxner A. Neurocognitive predictors of functional outcome two to 13 years after identification as ultra-high risk for psychosis. Schizophr Res. (2011) 132:1–7. 10.1016/j.schres.2011.06.014 21763109

[22] Chang WC Hui CLM Wong GHY Chan SKW Lee EHM Chen EYH. Symptomatic remission and cognitive impairment in first-episode schizophrenia:a prospective 3-year follow-up study. J Clin Psychiatry (2013) 74:e1046–53. 10.4088/JCP.13m08355 24330905

[23] Walder DJ Walker EF Lewine RJ. Cognitive functioning, cortisol release, and symptom severity in patients with schizophrenia. Biol. Psychiatry (2000) 48:1121–32. 10.1016/S0006-3223(00)01052-0 11137052

[24] Aas M Dazzan P Mondelli V Toulopoulou T Reichenberg A Di Forti M. Abnormal cortisol awakening response predicts worse cognitive function in patients with first-episode psychosis. Psychol Med. (2011) 41:463–76. 10.1017/S0033291710001170 20529412 PMC3513413

[25] Cullen AE Zunszain PA Dickson H Roberts RE Fisher HL Pariante CM. Cortisol awakening response and diurnal cortisol among children at elevated risk for schizophrenia:relationship to psychosocial stress and cognition. Psychoneuroendocrinology (2014) 46:1–13. 10.1016/j.psyneuen.2014.03.010 24882153 PMC4065330

[26] Allott KA Yuen HP Bartholomeusz CF Rapado-Castro M Phassouliotis C Butselaar F. Stress hormones and verbal memory in young people over the first 12 weeks of treatment for psychosis. Psychiatry Res. (2018) 260:60–6. 10.1016/j.psychres.2017.11.044 29175500

[27] Takahashi T Nakamura M Sasabayashi D Komori Y Higuchi Y Nishikawa Y. Olfactory deficits in individuals at risk for psychosis and patients with schizophrenia:relationship with socio-cognitive functions and symptom severity. Eur Arch Psychiatry Clin Neurosci. (2018) 268:689–98. 10.1007/s00406-017-0845-3 29071372

[28] Takahashi T Higuchi Y Komori Y Nishiyama S Nakamura M Sasabayashi D. Quality of life in individuals with attenuated psychotic symptoms:possible role of anxiety, depressive symptoms, and socio-cognitive impairments. Psychiatry Res. (2017) 257:431–7. 10.1016/j.psychres.2017.08.024 28837932

[29] Mizuno M Suzuki M Matsumoto K Murakami M Takeshi K Miyakoshi T. Clinical practice and research activities for early psychiatric intervention at Japanese leading centres. Early Interv Psychiatry (2009) 3:5–9. 10.1111/j.1751-7893.2008.00104.x 21352169

[30] Miyakoshi T Matsumoto K Ito F Ohmuro N Matsuoka H. Application of the Comprehensive Assessment of At-Risk Mental States (CAARMS) to the Japanese population:reliability and validity of the Japanese version of the CAARMS. Early Interv Psychiatry (2009) 3:123–30. 10.1111/j.1751-7893.2009.00118.x 21352185

[31] American Psychiatric Association Diagnostic and Statistical Manual of Mental Disorders. 4th ed. Text Revised. Washington DC: American Psychiatric Association (2000).

[32] First MB Gibbon M Spitzer RL Williams JBW Structured Clinical Interview for DSM-IV Axis I Disorders. Washington DC: American Psychiatric Press (1997).

[33] Kay SR Fiszbein A Opler LA. The positive and negative syndrome scale (PANSS) for schizophrenia. Schizophr Bull. (1987) 13:261–76. 10.1093/schbul/13.2.261 3616518

[34] Okada N Kasai K Takahashi T Suzuki M Hashimoto R Kameyama T Rating scale of handedness for biological psychiatry research among Japanese people. Japanese J Biol Psychiatry (2014) 25:118–9. 10.11249/jsbpjjpp.25.2_118

[35] Okada N Kasai K Takahashi T Suzuki M Hashimoto R Kawakami N Brief rating scale of socioeconomic status for biological psychiatry research among Japanese people:a scaling based on an educational history. Japanese J Biol Psychiatry (2014) 25:115–7. 10.11249/jsbpjjpp.25.2_115

[36] Matsuoka K Uno M Kasai K Koyama K Kim Y. Estimation of premorbid IQ in individuals with Alzheimer's disease using Japanese ideographic script (Kanji) compound words:Japanese version of national adult reading test. Psychiatry Clin Neurosci. (2006) 60:332–9. 10.1111/j.1440-1819.2006.01510.x 16732750

[37] Takahashi T Malhi GS Wood SJ Walterfang M Yücel M Lorenzetti V. Increased pituitary volume in patients with established bipolar affective disorder. Prog Neuropsychopharmacol Biol Psychiatry (2009) 33:1245–9. 10.1016/j.pnpbp.2009.07.012 19622379

[38] Takahashi T Suzuki M Velakoulis D Lorenzetti V Soulsby B Zhou SY. Increased pituitary volume in schizophrenia spectrum disorders. Schizophr Res. (2009) 108:114–21. 10.1016/j.schres.2008.12.016 19162445

[39] Malone IB Leung KK Clegg S Barnes J Whitwell JL Ashburner J. Accurate automatic estimation of total intracranial volume:a nuisance variable with less nuisance. Neuroimage (2015) 104:366–72. 10.1016/j.neuroimage.2014.09.034 25255942 PMC4265726

[40] Garner B Pariante CM Wood SJ Velakoulis D Phillips L Soulsby B. Pituitary volume predicts future transition to psychosis in individuals at ultra-high risk of developing psychosis. Biol Psychiatry (2005) 58:417–23. 10.1016/j.biopsych.2005.04.018 16026767

[41] Kaneda Y Sumiyoshi T Keefe R Ishimoto Y Numata S Ohmori T. Brief assessment of cognition in schizophrenia:validation of the Japanese version. Psychiatry Clin Neurosci. (2007) 61:602–9. 10.1111/j.1440-1819.2007.01725.x 18081619

[42] Keefe RS Goldberg TE Harvey PD Gold JM Poe MP Coughenour L. The brief assessment of cognition in schizophrenia:reliability, sensitivity, and comparison with a standard neurocognitive battery. Schizophr Res. (2004) 68:283–97. 10.1016/j.schres.2003.09.011 15099610

[43] Kaneda Y Ohmori T Okahisa Y Sumiyoshi T Pu S Ueoka Y Measurement and treatment research to improve cognition in schizophrenia consensus cognitive battery:validation of the Japanese version. Psychiatry Clin Neurosci. (2013) 67:182–8. 10.1111/pcn.12029 23581870

[44] Keefe RS Poe M Walker TM Kang JW Harvey PD. The schizophrenia cognition rating scale:an interview-based assessment and its relationship to cognition, real-world functioning, and functional capacity. Am J Psychiatry (2006) 163:426–32. 10.1176/appi.ajp.163.3.426 16513863

[45] Goldman HH Skodol AE Lave TR. Revising axis V for DSM-IV:a review of measures of social functioning. Am. J. Psychiatry (1992) 149:1148–56. 1386964 10.1176/ajp.149.9.1148

[46] American Psychiatric Association Diagnostic and Statistical Manual of Mental Disorders. 4th ed. Washington DC: American Psychiatric Association (1994).

[47] Walter A Studerus E Smieskova R Tamagni C Rapp C Borgwardt SJ. Pituitary gland volume in at-risk mental state for psychosis:a longitudinal MRI analysis. CNS Spectr. (2015) 20:122–9. 10.1017/S109285291400011X 24618395

[48] Pariante CM. Pituitary volume in psychosis:the first review of the evidence. J Psychopharmacol. (2008) 22:76–81. 10.1177/0269881107084020 18709702

[49] Cohrs S Röher C Jordan W Meier A Huether G Wuttke W The atypical antipsychotics olanzapine and quetiapine, but not haloperidol, reduce ACTH and cortisol secretion in healthy subjects. Psychopharmacology (2006) 185:11–8. 10.1007/s00213-005-0279-x 16432682

[50] Lurie SN Doraiswamy PM Husain MM Boyko OB Ellinwood EH Jr Figiel GS. *In vivo* assessment of pituitary gland volume with magnetic resonance imaging:the effect of age. J Clin Endocrinol Metab. (1990) 71:505–8. 2380345 10.1210/jcem-71-2-505

[51] MacMaster FP Keshavan M Mirza Y Carrey N Upadhyaya AR El-Sheikh R. Development and sexual dimorphism of the pituitary gland. Life Sci. (2007) 80:940–4. 10.1016/j.lfs.2006.11.040 17174342 PMC1853319

[52] Mondelli V Dazzan P Gabilondo A Tournikioti K Walshe M Marshall N. Pituitary volume in unaffected relatives of patients with schizophrenia and bipolar disorder. Psychoneuroendocrinology (2008) 33:1004–12. 10.1016/j.psyneuen.2008.05.010 18640787

[53] Gisselgård J Anda LG Brønnick K Langeveld J Ten Velden Hegelstad W Joa I. Verbal working memory deficits predict levels of auditory hallucination in first-episode psychosis. Schizophr Res. (2014) 153:38–41. 10.1016/j.schres.2013.12.018 24457037

[54] Pantelis C Stuart GW Nelson HE Robbins TW Barnes TR. Spatial working memory deficits in schizophrenia:relationship with tardive dyskinesia and negative symptoms. Am J Psychiatry (2001) 158:1276–85. 10.1176/appi.ajp.158.8.1276 11481163

[55] Huang J Tan SP Walsh SC Spriggens LK Neumann DL Shum DH. Working memory dysfunctions predict social problem solving skills in schizophrenia. Psychiatry Res. (2014) 220:96–101. 10.1016/j.psychres.2014.07.043 25110314

[56] Lett TA Voineskos AN Kennedy JL Levine B Daskalakis ZJ. Treating working memory deficits in schizophrenia:a review of the neurobiology. Biol Psychiatry (2014) 75:361–70. 10.1016/j.biopsych.2013.07.026 24011822

[57] Labad J Gutiérrez-Zotes A Creus M Montalvo I Cabezas Á Solé M. Hypothalamic-pituitary-adrenal axis measures and cognitive abilities in early psychosis:Are there sex differences? Psychoneuroendocrinology (2016) 72:54–62. 10.1016/j.psyneuen.2016.06.006 27344379

[58] Kamin HS Kertes DA. Cortisol and DHEA in development and psychopathology. Horm. Behav. (2017) 89:69–85. 10.1016/j.yhbeh.2016.11.018 27979632

[59] Harris DS Wolkowitz OM Reus VI. Movement disorder, memory, psychiatric symptoms and serum DHEA levels in schizophrenic and schizoaffective patients. World J Biol Psychiatry (2001) 2:99–102. 10.3109/15622970109027500 12587192

[60] Sánchez-Torres AM Elosúa MR Lorente-Omeñaca R Moreno-Izco L Cuesta MJ. A comparative study of the working memory multicomponent model in psychosis and healthy controls. Compr Psychiatry (2015) 61:97–105. 10.1016/j.comppsych.2015.05.008 26073063

[61] Keefe RS. The longitudinal course of cognitive impairment in schizophrenia:an examination of data from premorbid through posttreatment phases of illness. J Clin Psychiatry (2014) 75(Suppl. 2):8–13. 10.4088/JCP.13065su1.02 24919165

[62] Penadés R García-Rizo C Bioque M González-Rodríguez A Cabrera B Mezquida G. The search for new biomarkers for cognition in schizophrenia. Schizophr Res Cogn. (2015) 2:172–8. 10.1016/j.scog.2015.10.004 29114461 PMC5609637

